# Machine learning‐based prediction of meniscal tears in ACL reconstruction using BMI, time to surgery, injury mechanism, and Tegner activity score: A temporally validated decision tool

**DOI:** 10.1002/jeo2.70796

**Published:** 2026-05-25

**Authors:** Yushun Wu, Wenjing Luo, Fuwu Chen, Siyuan Lin, Weiquan Zeng, Jian Li

**Affiliations:** ^1^ Department of Sports Medicine The Second People's Hospital Affiliated to Fujian University of Traditional Chinese Medicine Fuzhou China; ^2^ The First Clinical College, Fujian University of Traditional Chinese Medicine Fuzhou China; ^3^ Department of Traditional Chinese Medicine Fuzhou Second General Hospital, Fuzhou Fujian China; ^4^ Department of Orthopedics Rehabilitation Hospital Affiliated to Fujian University of Traditional Chinese Medicine Fuzhou Fujian China

**Keywords:** anterior cruciate ligament reconstruction, clinical decision support, meniscal tears, preoperative prediction, temporal validation

## Abstract

**Purpose:**

Concomitant arthroscopically confirmed meniscal tears are common in patients undergoing anterior cruciate ligament (ACL) reconstruction and can influence intraoperative planning and postoperative rehabilitation. Robust tools for preoperative, individualized risk stratification remain limited. The objective of this study was to develop, temporally validate, and implement a clinically interpretable preoperative prediction tool for concomitant meniscal tears in ACL reconstruction.

**Methods:**

A retrospective analysis was conducted on 649 consecutive patients undergoing primary arthroscopic ACL reconstruction. Ten candidate machine‐learning algorithms were developed using routinely available preoperative variables. Model selection was performed via five‐fold cross‐validation in the development cohort. The selected model was evaluated in an internal validation set and an independent temporal validation cohort (comprising patients treated in a subsequent period to assess model stability). Discrimination (area under the receiver operating characteristic curve, AUC), calibration, and clinical utility (decision curve analysis) were assessed. Model interpretability was examined using SHapley Additive exPlanations (SHAP). An open‐access web calculator was created for point‐of‐care use.

**Results:**

Logistic regression using four routinely available preoperative predictors (body mass index, time from injury to surgery, injury mechanism and preoperative Tegner activity score) provided the most reliable performance. Discrimination remained consistent across cohorts (AUC 0.845 in training, 0.850 in internal validation, and 0.840 in temporal validation), with acceptable calibration. Decision curve analysis demonstrated a favourable net benefit across clinically relevant threshold probabilities. SHAP analyses supported the relative contribution and direction of effects of the four predictors. The final model was deployed as a web‐based calculator.

**Conclusions:**

An accurate, interpretable, and temporally validated preoperative prediction model for concomitant meniscal tears in ACL reconstruction was developed. By integrating four routine clinical variables into an online calculator, this tool may enhance surgical planning and inform shared decision‐making prior to ACL reconstruction.

**Level of Evidence:**

Level IV, retrospective cohort study.

## Graphical abstract

One‐sentence summary: A logistic regression model using BMI, time to surgery, injury mechanism, and Tegner score reliably predicted meniscal tears in ACL reconstruction and was temporally validated.

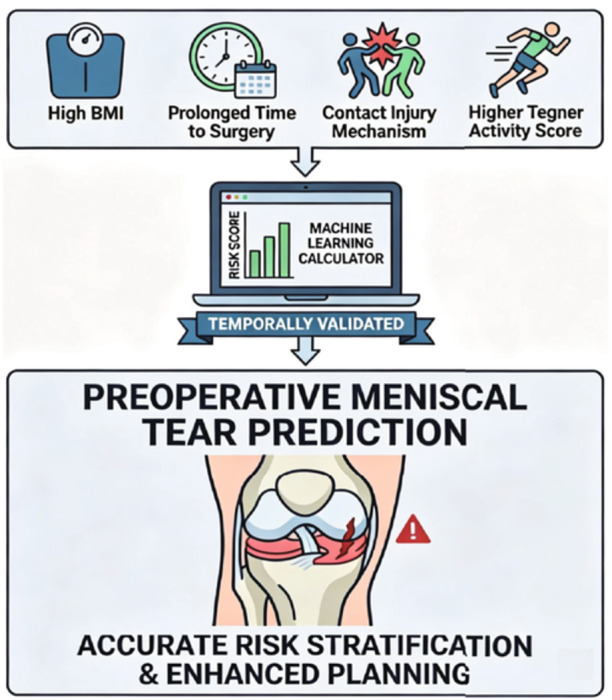

**Abbreviations**
ACL: anterior cruciate ligament
ACLR: anterior cruciate ligament reconstruction
AUC: area under the (receiver operating characteristic) curve
BMI: body mass index
CI: confidence interval
DCA: decision curve analysis
EPV: events per variable
ML: machine learning
ROC: receiver operating characteristic (curve)
SHAP: SHapley Additive exPlanations

## INTRODUCTION

Anterior cruciate ligament (ACL) injury is a common sports‐related trauma, for which reconstruction surgery is the standard treatment to restore knee stability [33]. Concomitant meniscal tears are frequently encountered during the time of Anterior cruciate ligament reconstruction (ACLR)—with a reported prevalence ranging from 40% to 70%—and significantly influence intraoperative strategies (repair vs. partial meniscectomy), postoperative rehabilitation, and long‐term joint health [3, 7, 26]. Accordingly, accurate preoperative anticipation of meniscal tears can directly improve surgical preparedness, such as ensuring the availability of repair‐specific instrumentation, refine patient counselling, refine patient counselling and support individualized perioperative planning [22, 28, 32].

Despite this clinical necessity, reliable preoperative risk stratification remains challenging. Physical examination demonstrates limited specificity in the acute setting, while magnetic resonance imaging (MRI) remains the gold standard for noninvasive diagnosis, it can demonstrate variable diagnostic performance for subtle or complex tears, and interpretation may be subject to inter‐observer variability. Furthermore, even with clear preoperative imaging, a recognized ‘gap’ exists in translating static findings into an individualized, quantitative risk estimate that can be directly operationalized in surgical decision‐making [1]. In clinical practice, multiple factors—including body habitus, injury mechanism, and time from injury—must be synthesized. Notably, A large‐scale study involving 4697 patients, recently quantified key risk factors for medial meniscal lesions at the time of ACL reconstruction, confirming that BMI, time to surgery, and sex are independent predictors [9]. However, there remains a lack of validated tools to integrate these variables into an objective probability estimate to guide the repair‐versus‐resection decision [5].

Clinical prediction modelling can address this gap by synthesizing routine preoperative data into individualized risk estimates [24, 30]. Although machine learning (ML) applications in orthopedics are expanding, several critical gaps remain in the development of preoperative prediction models specifically for concomitant meniscal tears in ACLR patients. Existing studies are often limited by modest sample sizes, which increases the risk of overfitting and limits generalizability; [2] few have undergone rigorous temporal validation—testing on a subsequent, independent patient cohort—which is essential to assess reliability as clinical populations and practices evolve [27]. Additionally, many models function as ‘black boxes’ with limited interpretability, hindering clinical trust and adoption [35]. Critically, there is a lack of deployable tools that translate model predictions into practical, point‐of‐care decision support for surgeons [27, 35]. For surgical decision support, model robustness, transparency and demonstrated clinical utility are as crucial as discrimination metrics alone.

Therefore, this study aimed to develop, temporally validate, and implement an interpretable preoperative decision‐support tool for arthroscopically confirmed meniscal tears in ACLR patients. It was hypothesized that a model based on a restricted set of routinely available preoperative variables would accurately predict the presence of concomitant meniscal tears and maintain its predictive performance on a temporally distinct patient cohort, thereby providing a practical tool to support preoperative risk stratification and surgical planning. By utilizing routinely available preoperative variables, multiple ML algorithms were compared, and an optimal model was selected via cross‐validation. Performance was comprehensively evaluated in terms of discrimination, calibration, and—importantly—clinical utility via decision curve analysis (DCA) to provide an objective adjunct to traditional diagnostic protocols, thereby enhancing preoperative planning and patient counselling. Model transparency was enhanced using SHapley Additive exPlanations (SHAP), and the validated model was ultimately translated into an open‐access, web‐based calculator to facilitate immediate integration into the clinical workflow.

## MATERIALS AND METHODS

### Study design and participants

**Figure 1:**
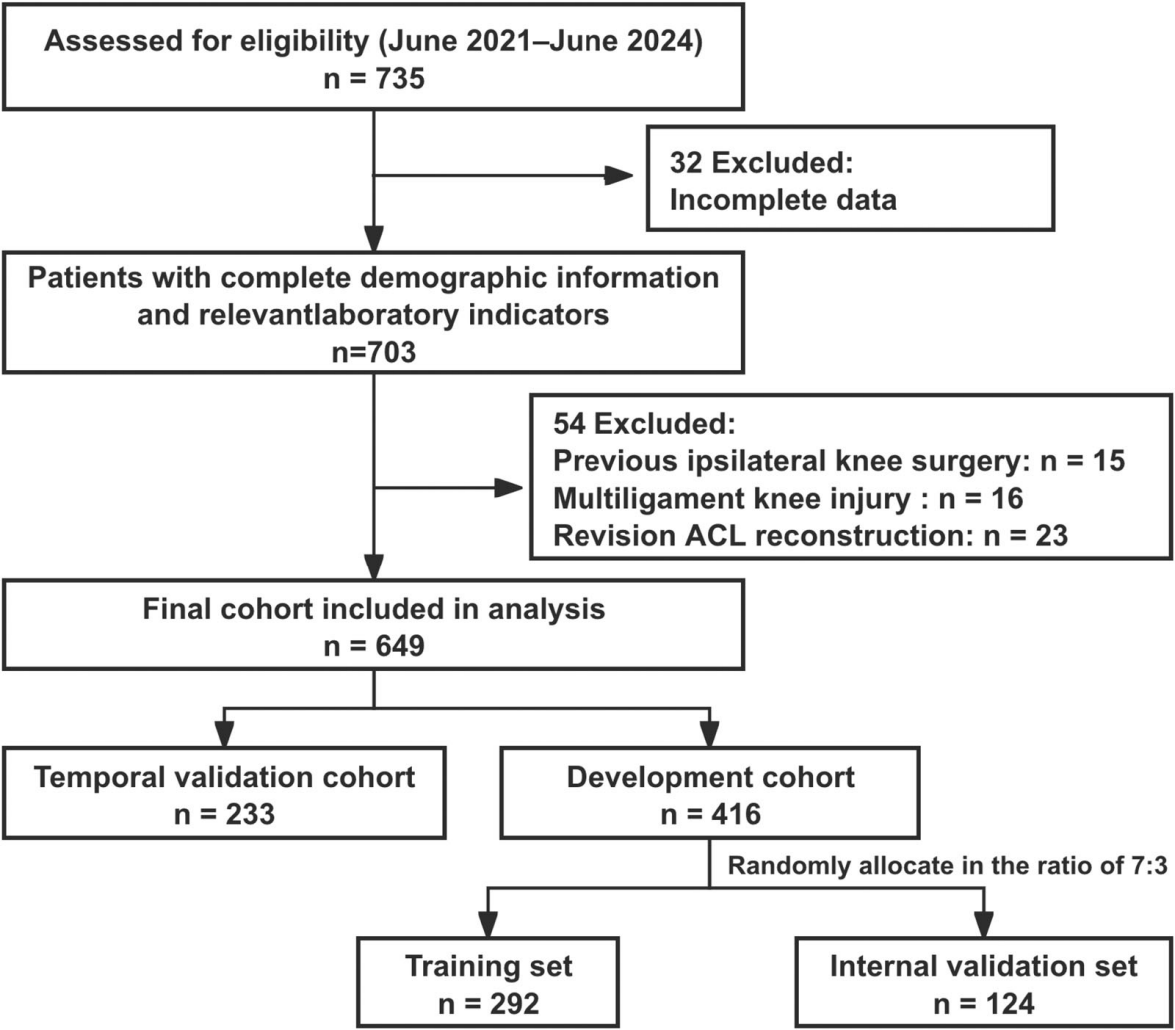
Trial flow diagram.

This retrospective cohort study was conducted at a single Grade A tertiary hospital in China and included consecutive patients who underwent primary arthroscopic ACLR between June 2021 and June 2024. The inclusion criteria were defined as: (1) a confirmed diagnosis of ACL rupture [13], (2) primary arthroscopic ACL reconstruction, and (3) availability of complete preoperative clinical data. Exclusion criteria comprised previous ipsilateral knee surgery, multiligament knee injuries (grade III or higher), and revision ACL reconstruction. A complete case analysis was performed; patients with any missing data regarding the candidate predictors or the primary outcome were excluded from the study population. The trial flow diagram is shown in Figure 1.

The study protocol was reviewed and approved by the institutional ethics committee (Approval Code: SPHFJP‐T2024031‐02 and Approval Date: September 18, 2024). The requirement for informed consent was waived due to the retrospective nature of the study, which involved analysis of anonymized data [18].

### Outcome and predictors

The primary outcome was the intraoperative confirmation of a concomitant meniscal tear (medial, lateral or both) [11], as documented in the surgical report. Candidate predictors were selected a priori based on clinical relevance and existing literature. Given the stringent inclusion criteria, no imputation methods were required, as only patients with a full complement of preoperative variables were analysed. The analyzed variables encompassed body mass index (BMI) [14], time from injury to surgery (in weeks) [6], injury mechanism (categorized as contact or noncontact) [27], preoperative Tegner activity score [2], and other baseline demographic and clinical characteristics, including age, sex and the presence of bone contusions on MRI.

### Data partitioning and validation strategy

To simulate real‐world clinical deployment and ensure the model's reliability for future patients, a stringent temporal validation strategy was employed. This method tests the model on patients treated in a subsequent time period, providing the most clinically relevant estimate of its performance when applied prospectively in practice [23]. The entire cohort was partitioned into two mutually exclusive sets based strictly on the date of surgery, ensuring that no patients from the later time period influenced model development in any way:

Development Cohort: Patients who underwent surgery between June 2021 and December 2023 (*n* = 416). This cohort was used for all stages of model development, including feature selection, algorithm training, and hyperparameter tuning. To monitor performance during development and prevent overfitting, this cohort was further randomly split into a training set (70%, *n* = 292) for model building and hyperparameter tuning, and an internal validation set (30%, *n* = 124) for cross‐validation monitoring [40]. Data from the temporal validation cohort were never accessed or used during any phase of model development.

Temporal Validation Cohort: Patients who underwent surgery between January 2024 and June 2024 (*n* = 233). This cohort consisted of patients treated in a subsequent, nonoverlapping time period and was completely held out and isolated during the entire model development process. It served as an independent test set to provide a final, unbiased estimate of model performance on unseen data from a subsequent time period [31]. Performance on this cohort reflects how the model would perform when deployed prospectively in clinical practice on new patients, offering a more rigorous test than internal validation alone.

### Model development and selection

Predictive models were constructed and compared using the tidymodels framework in R (version 4.2.1) [19]. To optimize model parsimony and minimize overfitting, a two‐stage predictor selection process was performed within the training cohort. Initially, all candidate variables were screened using univariate logistic regression. Variables demonstrating a significant association (*p* < 0.05) were then entered into a multivariable logistic regression model to identify independent predictors. Ten supervised ML algorithms were implemented, including logistic regression, LASSO regression, ridge regression, decision tree, random forest, extreme gradient boosting (XGBoost), support vector machine, k‐nearest neighbours and multilayer perceptron [34, 38]. Model training and hyperparameter tuning were conducted using a five‐fold cross‐validation procedure repeated on the training set, with the area under the receiver operating characteristic curve (AUC) as the primary optimization metric [10, 34]. Detailed performance metrics and tuning outcomes for all candidate algorithms across the training, internal validation, and temporal validation cohorts are explicitly summarized in Table S1. The model that demonstrated the best and most consistent cross‐validation performance was selected as the final model.

### Model evaluation

To ensure the robustness of the machine‐learning models, sample size adequacy was assessed using the Events Per Variable (EPV) criterion. With 220 meniscal tear events in the training set and 4 final candidate predictors, an EPV of 55 was achieved. This ratio is substantially higher than the traditional threshold of 10–15 EPV recommended for stable clinical prediction modelling, thereby minimizing the risk of overfitting and ensuring the reliability of the estimated parameters. The performance of the final model was comprehensively evaluated across the training set, internal validation set, and temporal validation set. Evaluation focused on three key dimensions: Discrimination: Assessed using the AUC and its 95% confidence interval (CI), visualized by receiver operating characteristic (ROC) curves [37]. Calibration: Assessed using calibration plots to evaluate the agreement between predicted probabilities and observed event rates. While the Hosmer–Lemeshow (HL) test was also performed, its limitations—including sensitivity to sample size and potential overfitting in smaller datasets—were acknowledged [15]. Clinical Utility: Assessed using DCA to quantify the net clinical benefit across a range of clinically reasonable risk thresholds [16].

### Model interpretation and deployment

To explain the model predictions and enhance clinical interpretability, SHAP were applied to quantify the contribution and directional effect of each predictor [17]. Based on the final model parameters, an open‐access, web‐based clinical calculator was developed using the Shiny framework for R and deployed online to facilitate point‐of‐care risk assessment [39]. The calculator is available at https://nemn.shinyapps.io/dynnomapp-1/.

## RESULTS

### Patient characteristics and cohort partitioning

A total of 649 patients who underwent ACLR were included in the final analysis. The entire cohort was divided into a training cohort (*n* = 292), an internal validation cohort (*n* = 124), and a temporal validation cohort (*n* = 233) using a strict temporal split. In the training cohort, the prevalence of meniscal tears was 75.3% (*n* = 220), while the internal validation and temporal validation cohorts showed prevalence rates of 80.6% and 60.1%, respectively. This relatively high prevalence of concomitant injury provided a robust dataset for model training, ensuring that the machine‐learning algorithms could effectively learn the patterns associated with meniscal pathology. The observed sex imbalance across the cohorts (65.3% male) is consistent with the established epidemiology of ACL ruptures within the surgical population of the studied institution, reflecting a higher volume of male patients undergoing reconstruction during the study period. Baseline demographic and clinical characteristics across the three cohorts are summarized in Table 1.

**Table 1:** Baseline demographic and clinical characteristics of the cohorts.

	Temporal validation	Internal validation	Train	Total	*p* Value
Total	233	124	292	649	
Sex					<0.001
Male	111 (47.6)	89 (71.8)	224 (76.7)	424 (65.3)	
Female	122 (52.4)	35 (28.2)	68 (23.3)	225 (34.7)	
Age					0.235
Median (IQR)	31 (21, 41)	29.5 (21.8, 35)	29 (22, 36)	30 (22, 38)	
BMI					0.769
Mean (SD)	25.4 (3.4)	25.2 (3.1)	25.2 (3.2)	25.2 (3.3)	
Time from injury to surgery					<0.001
Median (IQR)	17 (9, 25)	4 (2, 24)	5 (2, 25)	9 (3, 25)	
Injury mechanism					<0.001
Contact	103 (44.2)	28 (22.6)	70 (24)	201 (31)	
Noncontact	130 (55.8)	96 (77.4)	222 (76)	448 (69)	
Preoperative tegner					0.96
Median (IQR)	4 (2,5)	4 (3, 4)	4 (3, 4)	4 (3, 5)	
Preoperative IKDC					0.306
Median (IQR)	38 (28, 50)	38 (30, 40)	38.5 (30, 45.2)	38 (30, 45)	
Preoperative Lysholm					0.026
Median (IQR)	43 (32, 53)	45.5 (36, 50)	45 (40, 55)	45 (35, 53)	
Segond fracture					0.257
No	227 (97.4)	122 (98.4)	280 (95.9)	629 (96.9)	
Yes	6 (2.6)	2 (1.6)	12 (4.1)	20 (3.1)	
Kissing contusion					<0.001
No	119 (51.1)	100 (80.6)	228 (78.1)	447 (68.9)	
Yes	114 (48.9)	24 (19.4)	64 (21.9)	202 (31.1)	
Meniscal tear					<0.001
No	93 (39.9)	24 (19.4)	72 (24.7)	189 (29.1)	
Yes	140 (60.1)	100 (80.6)	220 (75.3)	460 (70.9)	

*Note*: Data are presented as *n* (%) for categorical variables and as mean ± standard deviation (SD) or median (interquartile range, IQR) for continuous variables, as appropriate. *p* Values were derived from chi‐square tests for categorical variables and either analysis of variance (ANOVA) or Kruskal–Wallis tests for continuous variables to assess differences across the three cohorts.

Abbreviation: BMI, body mass index.

### Identification of independent predictors

Predictor selection was conducted in a two‐stage process within the training cohort. First, univariate logistic regression analyses were performed to assess the crude association between each candidate variable and the presence of meniscal tears (Table 2). Variables demonstrating a significant association (*p* < 0.05) were then entered into a multivariable logistic regression model to identify independent predictors while adjusting for other factors.

**Table 2:** Univariate logistic regression analysis of factors associated with meniscal tears in the training cohort.

Characteristics	Univariate logistic analysis		
	OR	95% CI	*p* value
Sex	0.417	0.232–0.751	0.004
Age	0.968	0.944–0.992	0.01
BMI	1.349	1.21–1.504	0.001
Time from injury to surgery	1.029	1.01–1.049	0.003
Injury mechanism	5.99	3.314–10.829	0.001
Preoperative Tegner	1.913	1.457–2.51	0.001
Preoperative IKDC	0.984	0.961–1.007	0.178
Preoperative Lysholm	0.983	0.959–1.008	0.191
Segond fracture	0.44	0.135–1.433	0.173
Kissing contusion	0.65	0.352–1.199	0.168

*Note*: Variables with a *p* < 0.05 were considered statistically significant and were selected for inclusion in the subsequent multivariable analysis.

Abbreviations: BMI, body mass index; CI, confidence interval; OR, adjusted odds ratio.

The results of the final multivariable model are presented in Table 3. Four preoperative variables remained independently associated with an increased risk of meniscal tears: higher body mass index (BMI), a longer time from injury to surgery, a contact injury mechanism (vs. noncontact), and a higher preoperative Tegner activity score (all *p* < 0.05). These four predictors were subsequently used for the development of all ML models.

**Table 3:** Multivariable logistic regression model for independent predictors of meniscal tears in the training cohort.

Characteristics	Multivariate logistic analysis		
	OR	95% CI	*p* value
Sex	1.18	0.524–2.657	0.69
Age	0.981	0.951–1.011	0.213
BMI	1.339	1.177–1.523	0.001
Time from injury to surgery	1.021	1.006–1.037	0.007
Injury mechanism	5.509	2.669–11.373	0.001
Preoperative Tegner	1.814	1.313–2.505	0.001

*Note*: The model was constructed using the six variables that showed significance in the univariate analysis.

Abbreviations: BMI, body mass index; CI, confidence interval; OR, adjusted odds ratio.

Notably, while male sex was associated with an increased risk of tears in univariate analysis (OR = 0.417), its effect was attenuated and the OR direction reversed in the multivariable model (OR = 1.18, *p* = 0.69). This shift suggests that the influence of sex was largely confounded by other clinical factors, such as higher BMI and contact injury mechanisms, which were more strongly and independently associated with meniscal pathology.

### Model performance comparison

Ten supervised ML algorithms were developed and compared. Their discriminative performance, as measured by the area under the ROC curve (AUC), across the training, internal validation, and temporal validation cohorts is summarized in Table 4 (detailed metrics are available in Table S1).

**Table 4:** Performance comparison of machine learning algorithms for predicting meniscal tears across different cohorts.

Algorithm (Abbreviation)	Training cohort AUC	Internal validation cohort AUC	Temporal validation cohort AUC	Mean AUC	SD of AUC
Decision Tree (DT)	0.898	0.861	0.605	0.788	0.15956
Random Forest (RF)	0.938	0.895	0.785	0.8727	0.07891
XGBoost	0.865	0.792	0.711	0.7893	0.07703
lightgbm	0.992	0.892	0.777	0.887	0.10759
SVM	0.841	0.828	0.844	0.8377	0.0085
MLP	0.818	0.798	0.859	0.825	0.0311
KNN	0.92	0.717	0.814	0.817	0.10153
Logistic Regression (LR)	0.845	0.85	0.84	0.845	0.005
Lasso	0.824	0.82	0.856	0.8333	0.01973
Ridge	0.831	0.827	0.861	0.8397	0.01858

*Note*: Performance is primarily evaluated by the AUC.

Abbreviations: AUC, area under the receiver operating characteristic curve; SD, standard deviation.

Across the three datasets, the logistic regression model demonstrated stable and consistent discriminative performance, with AUC values of 0.845 in the training cohort, 0.850 in the internal validation cohort, and 0.840 in the temporal validation cohort. Other models, including support vector machine and ridge regression, also showed acceptable discrimination. However, several complex ML models, such as random forest and LightGBM, exhibited substantially reduced AUC values in the temporal validation cohort compared to the training and internal validation cohorts, suggesting potential overfitting.

**Figure 2:**
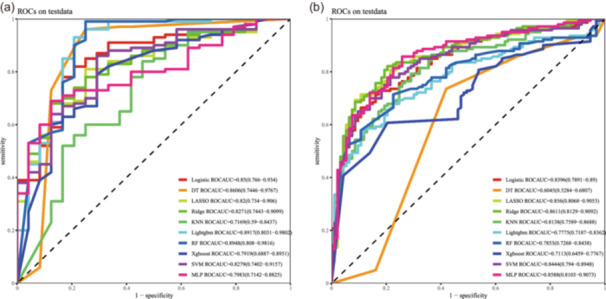
Receiver operating characteristic (ROC) curves for model discrimination in the validation cohorts. (a) ROC curves of the ten candidate models in the internal validation cohort. (b) ROC curves of the ten candidate models in the temporal validation cohort. The *y*‐axis represents the true positive rate (Sensitivity), and the *x*‐axis represents the false positive rate (1‐Specificity). The comparison highlights that the logistic regression model maintained the most stable AUC across both validation sets.

The superior and consistent performance of the optimal logistic regression model in the independent validation cohorts is visually confirmed in Figure 2. The ROC curves show that the model maintains high discriminatory ability in both the internal and temporal validation sets, with AUC values aligning closely with those reported in Table 4. This visual evidence underscores model robustness and generalizability beyond the development data.

### Model calibration and clinical utility

**Figure 3:**
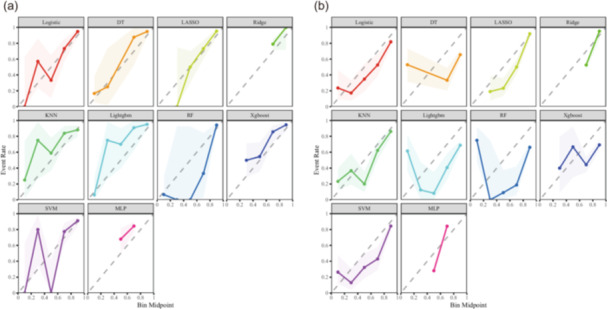
Calibration curves for model discrimination in the validation cohorts. (a) Calibration curves in the internal validation cohort. (b) Calibration curves in the temporal validation cohort. The diagonal dashed line represents perfect calibration.

Calibration curves for all models are shown in Figure 3. The logistic regression model demonstrated acceptable agreement between predicted probabilities and observed outcomes across all cohorts. Consistent calibration was exhibited compared with other models, particularly in the temporal validation cohort.

**Figure 4:**
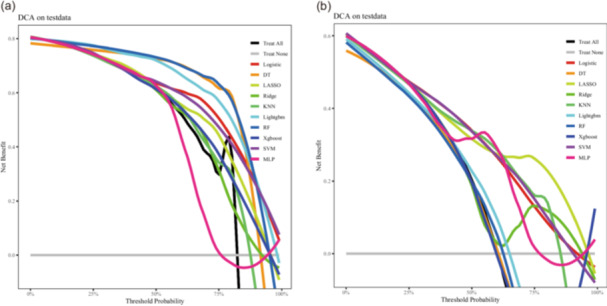
Decision curve analysis (DCA) for the candidate models in the validation cohorts. (a) DCA in the internal validation cohorts; (b) DCA in the temporal validation cohorts. The *y*‐axis indicates the Net Benefit, while the *x*‐axis represents the risk threshold probability.

To assess the clinical utility of the models, DCA was performed. As shown in Figure 4, the logistic regression model yielded the highest net clinical benefit across a wide range of threshold probabilities in all three cohorts, indicating its superior clinical usefulness for preoperative risk assessment. Model calibration remained stable despite prevalence differences. In the temporal validation cohort, the model demonstrated a Brier score of 0.183, a calibration slope of 0.88 (95% CI: 0.74–1.02), and an intercept of 0.31 (95% CI: 0.02–0.60, *p* = 0.04), indicating acceptable agreement between predicted and observed probabilities.

Based on a comprehensive evaluation of discrimination, calibration, and clinical utility, the logistic regression model was selected as the optimal predictive model.

### Model interpretation using SHAP

**Figure 5:**
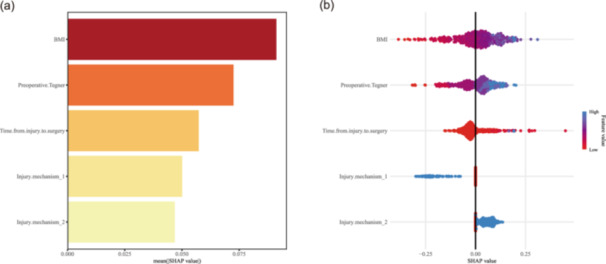
SHapley Additive exPlanations (SHAP) analysis for the final logistic regression model. (a) SHAP summary bar plot ranking the four predictors by their mean absolute SHAP value, indicating each variable's overall contribution to the model output. Higher values denote greater influence on predicted risk. (b) SHAP summary dot plot showing the direction and magnitude of each variable's effect. Each dot represents an individual patient; colour indicates the variable value (red: high, blue: low). Positive SHAP values (right side) increase the predicted probability of a meniscal tear.

To improve model transparency and interpretability, SHAP were applied to the final logistic regression model. BMI emerged as the most influential factor (mean SHAP = 0.091), followed by the preoperative Tegner score (mean SHAP = 0.072), time from injury to surgery (mean SHAP = 0.058), and injury mechanism (mean SHAP = 0.045). The SHAP summary bar plot (Figure 5a) ranks these predictors by their overall contribution, while the SHAP summary dot plot (Figure 5b) further illustrates the direction and magnitude of each predictor effect on meniscal tear risk. Higher BMI, higher preoperative Tegner score, and longer time from injury to surgery were associated with an increased predicted risk of meniscal tears. Additionally, specific injury mechanisms were linked to a higher probability of meniscal tears.

### Web‐based model deployment

To facilitate clinical application, the optimal logistic regression model was implemented as an open‐access, web‐based risk calculator. This interactive tool allows clinicians to input specific patient data—including BMI, injury mechanism, time from injury, and Tegner activity score—to generate a personalized, quantitative probability of a concomitant meniscal tear. As a clinical decision support tool, the calculator provides an immediate risk estimate designed to augment traditional diagnostic modalities, such as MRI, and assist in preoperative counselling. Notably, the tool is intended to function as a practical adjunct to, rather than a replacement for, the comprehensive clinical judgement and multi‐modal diagnostic evaluation performed by the surgeon.

## DISCUSSION

In this study, a clinical tool for the preoperative assessment of concomitant meniscal tear risk in patients undergoing ACLR was developed and temporally validated. The findings demonstrate that a parsimonious logistic regression model provides robust discrimination and acceptable calibration across chronologically distinct cohorts. By synthesizing readily available clinical variables into a quantitative risk estimate, this model offers a data‐driven adjunct to augment traditional diagnostic assessments and surgical expertise.

Importantly, the developed model is not intended to supplant the diagnostic role of MRI or the intraoperative judgment of the surgeon; rather, it is designed to function as a clinical decision support system. While experienced surgeons are generally prepared for meniscal pathology, the primary value of this preoperative tool lies in two clinical scenarios. First, enhanced patient counselling is facilitated through a shared decision‐making process. For high‐risk patients, the implications of meniscal repair—such as restricted weight‐bearing and prolonged rehabilitation—can be preemptively discussed, thereby managing expectations more effectively than via a general intraoperative contingency. Second, logistical optimization is supported. Accurate risk stratification assists in surgical scheduling and resource allocation, ensuring that high‐risk cases are allotted sufficient theater time and prioritized for specialized equipment or surgical assistants, ultimately improving operative efficiency.

The four predictors incorporated into the final model are grounded in established biomechanical and clinical principles. Elevated BMI is associated with greater mechanical load across the knee, potentially exacerbating meniscal stress after ACL injury [9, 36]. A longer interval from injury to surgery has been associated with a higher probability of secondary meniscal damage, plausibly reflecting ongoing instability and repetitive microtrauma in the ACL‐deficient knee [7, 8, 25, 35]. The mechanism of injury encodes the direction and magnitude of traumatic force, which is directly relevant to the pattern of intra‐articular pathology [4]. Mechanistically, a higher preoperative Tegner score reflects involvement in high‐intensity, pivoting sports. High‐energy injury mechanisms in these active individuals are more likely to result in significant rotatory instability, thereby increasing the risk of concomitant meniscal damage compared to low‐demand individuals [16]. The present model synthesizes these factors into a single, interpretable risk score, advancing beyond the consideration of isolated risk factors.

Notably, age did not emerge as a significant independent predictor, which may appear counterintuitive given its association with meniscal degeneration. However, this finding is likely attributable to the homogeneity of the present cohort—primarily young, active patients undergoing primary ACLR—where lesions are predominantly acute and traumatic. In this population, the predictive power of mechanical indicators, such as Segond fractures and kissing contusions, likely overshadowed chronological age, as these markers more directly reflect the high‐energy mechanism of the index injury.

A pivotal methodological insight was the superior clinical reliability of the simpler logistic regression model. Although complex algorithms such as random forest and gradient boosting achieved high performance during development, their discriminative ability notably declined upon temporal validation, indicating limited generalizability. In contrast, logistic regression demonstrated stable performance. Beyond statistical robustness, this model was selected for its inherent clinical virtues: easily interpretable odds ratios are generated, fostering immediate trust and understanding among surgeons, and its computational simplicity allows for seamless integration into a real‐time web calculator. For a surgical decision aid, this transparency and practical efficiency are paramount, often outweighing marginal gains in accuracy from less interpretable ‘black‐box’ alternatives [20, 21].

While many models over‐rely on the AUC, priority was placed on calibration, which determines the accuracy of the specific risk percentage presented to the patient. Despite the prevalence shift in our population, quantitative calibration remained robust, ensuring that the probabilities generated by our web‐based tool serve as reliable clinical signals. Furthermore, DCA demonstrated a positive net benefit across a broad range of threshold probabilities (10% to 40%). This confirms that the use of the predicted probabilities generated by the model allows for higher clinical utility than empirical judgment alone, effectively balancing the risk of missed tears against the cost of unnecessary surgical preparations.

To bridge the gap between model development and clinical adoption, SHAP were utilized to quantify and visualize the contribution of each predictor [12]. This transformed the model into a transparent decision aid, empowering surgeons to discuss modifiable and nonmodifiable risks with patients. To further enhance translation into routine care, the final model was deployed as a publicly accessible web calculator. This tool is designed for seamless integration into clinical workflows, requiring only routine preoperative data to generate an individualized risk assessment.

Several limitations should be considered. First, the retrospective, single‐centre design may introduce selection bias. While the sample (*n* = 649) is substantial, it may be considered borderline for certain ML architectures. However, a robust EPV ratio of 55 (220 events/4 variables) was yielded in the training cohort, far exceeding the threshold for stable modelling. Second, a prevalence shift in meniscal tears between the development cohort (75.3%) and the temporal validation cohort (60.1%) was observed. Notably, while the calibration slope remained close to unity—indicating that the relative ranking of predicted risks was preserved—the calibration intercept of 0.31 (*p* = 0.04) confirmed a systematic overestimation of the absolute risk. This discrepancy implies that, although the four predictors (BMI, time to surgery, injury mechanism, and Tegner activity score) retained their discriminative value across cohorts, the model tended to overstate the predicted probability of a meniscal tear, which could lead to unnecessary preoperative anxiety or over‐preparation of surgical resources if used without recalibration. Third, the retrospective nature of the study precluded the inclusion of specific dynamic markers, such as pivot‐shift or rotational instability assessments. As highlighted by Sakamoto et al. these variables are independent predictors of medial meniscal tears and their absence represents a study limitation [29]. Furthermore, LASSO regression was utilized rather than recursive feature elimination to prioritize a clinically accessible feature set. Future multi‐center, prospective studies are required to externally validate these findings and incorporate standardized physical examinations to further refine the model's predictive depth.

## CONCLUSIONS

In conclusion, a parsimonious, clinically‐driven model for predicting concomitant meniscal tears during ACL reconstruction was validated. By synthesizing BMI, injury mechanism, time from injury to surgery, and preoperative activity levels, the model maintained high discriminative power and acceptable calibration despite temporal shifts in injury prevalence. The integration of SHAP‐based interpretability and a publicly accessible web calculator bridges the gap between ML complexity and bedside utility. Ultimately, this tool serves as a reliable, data‐driven adjunct to augment surgical scheduling, patient counselling, and shared decision‐making in sports medicine.

## AUTHOR CONTRIBUTIONS

Yushun Wu contributed to the study design, data collection and analysis, and manuscript draughting and revision. Wenjing Luo was responsible for statistical analysis, software development, model validation, and manuscript writing. Fuwu Chen participated in data collection, statistical analysis and manuscript revision. Siyuan Lin participated in data collection, statistical analysis and manuscript revision. Weiquan Zeng contributed to the study design, coordination, and critical revision of the manuscript. Jian Li contributed to the study design, coordination, and critical revision of the manuscript. All authors reviewed and approved the final version of the manuscript.

## CONFLICT OF INTEREST STATEMENT

The authors declare no conflict of interest.

## ETHICS STATEMENT

This study was conducted in accordance with the guidelines of the ‘Declaration of Helsinki’ and was approved by the ethics committee of our hospital (Approval Code: SPHFJP‐T2024031‐02 and Approval Date: 18 September 2024). All the data were analysed anonymously, and personal identifiers were completely deleted. The requirement for informed consent was waived due to the retrospective nature of the study, which involved analysis of anonymized data.

## Supporting information


**Supplementary Table S1.** Performance Comparison of Machine Learning Algorithms for Predicting Meniscal Tears Across Different Cohorts. Abbreviations: AUC, area under the receiver operating characteristic curve. Note: This table provides detailed performance metrics for the ten machine learning algorithms evaluated in this study, including the area under the receiver operating characteristic curve (AUC), accuracy, sensitivity, specificity, F1‐score, and Brier score. Results are reported separately for the training cohort (n = 291), internal validation cohort (n = 125), and temporal validation cohort (n = 233). These metrics provide the empirical basis for hyperparameter tuning and model selection, complementing the comparative overview presented in the main text.

## Data Availability

The datasets generated and analyzed during the current study are not publicly available due to patient privacy regulations but are available from the corresponding author upon reasonable request.
