# Supplementary material for: Machine learning‐based prediction of meniscal tears in ACL reconstruction using BMI, time to surgery, injury mechanism, and Tegner activity score: A temporally validated decision tool
Source: J Exp Orthop. 2026 May 25;13(2):e70796. doi: 10.1002/jeo2.70796 (PMC13239857; doi:10.1002/jeo2.70796)
Supplement: Supplementary file 1 — Supplementary Table S1. Performance Comparison of Machine Learning Algorithms for Predicting Meniscal Tears Across Different Cohorts. Abbreviations: AUC, area under the receiver operating characteristic curve. Note: This table provides detailed performance metrics for the ten machine learning algorithms evaluated in this study, including the area under the receiver operating characteristic curve (AUC), accuracy, sensitivity, specificity, F1‐score, and Brier score. Results are reported separately for the training cohort (n = 291), internal validation cohort (n = 125), and temporal validation cohort (n = 233). These metrics provide the empirical basis for hyperparameter tuning and model selection, complementing the comparative overview presented in the main text. [file JEO2-13-e70796-s001.docx]

**Supplementary Table S1.** Performance Comparison of Machine Learning Algorithms for Predicting Meniscal Tears Across Different Cohorts.

| **Algorithm (Abbreviation)** | **Training Cohort** | | | | | | **Internal Validation Cohort** | | | | | | **Temporal Validation Cohort** | | | | | |
| --- | --- | --- | --- | --- | --- | --- | --- | --- | --- | --- | --- | --- | --- | --- | --- | --- | --- | --- |
|  | **AUC** | **accuracy** | **sensitivity** | **specificity** | **F1-score** | **Brier score** | **AUC** | **accuracy** | **sensitivity** | **specificity** | **F1-score** | **Brier score** | **AUC** | **accuracy** | **sensitivity** | **specificity** | **F1-score** | **Brier score** |
| Decision Tree（DT） | 0.898 | 0.89 | 0.936 | 0.75 | 0.928 | 0.0728 | 0.861 | 0.871 | 0.89 | 0.792 | 0.918 | 0.0795 | 0.605 | 0.644 | 0.857 | 0.323 | 0.743 | 0.321 |
| Random Forest（RF） | 0.938 | 0.839 | 0.818 | 0.903 | 0.885 | 0.0903 | 0.895 | 0.726 | 0.7 | 0.833 | 0.805 | 0.0667 | 0.785 | 0.717 | 0.679 | 0.774 | 0.742 | 0.319 |
| XGBoost | 0.865 | 0.753 | 0.723 | 0.847 | 0.815 | 0.12 | 0.792 | 0.597 | 0.53 | 0.875 | 0.679 | 0.139 | 0.711 | 0.635 | 0.721 | 0.505 | 0.704 | 0.269 |
| lightgbm | 0.992 | 0.973 | 0.977 | 0.958 | 0.982 | 0.0385 | 0.892 | 0.847 | 0.86 | 0.792 | 0.901 | 0.0778 | 0.777 | 0.657 | 0.907 | 0.28 | 0.76 | 0.292 |
| SVM | 0.841 | 0.791 | 0.805 | 0.75 | 0.853 | 0.124 | 0.828 | 0.782 | 0.8 | 0.708 | 0.856 | 0.117 | 0.844 | 0.773 | 0.793 | 0.742 | 0.807 | 0.181 |
| MLP | 0.818 | 0.695 | 0.641 | 0.861 | 0.76 | 0.183 | 0.798 | 0.645 | 0.58 | 0.917 | 0.725 | 0.178 | 0.859 | 0.725 | 0.586 | 0.935 | 0.719 | 0.201 |
| KNN | 0.92 | 0.812 | 0.782 | 0.903 | 0.862 | 0.0971 | 0.717 | 0.694 | 0.72 | 0.583 | 0.791 | 0.0728 | 0.814 | 0.738 | 0.729 | 0.753 | 0.77 | 0.0728 |
| Logistic Regression（LR） | 0.845 | 0.788 | 0.795 | 0.764 | 0.85 | 0.123 | 0.85 | 0.831 | 0.86 | 0.708 | 0.891 | 0.114 | 0.84 | 0.755 | 0.793 | 0.699 | 0.796 | 0.183 |
| Lasso | 0.824 | 0.729 | 0.695 | 0.833 | 0.795 | 0.14 | 0.82 | 0.702 | 0.68 | 0.792 | 0.786 | 0.127 | 0.856 | 0.773 | 0.693 | 0.892 | 0.785 | 0.175 |
| Ridge | 0.831 | 0.747 | 0.718 | 0.833 | 0.81 | 0.168 | 0.827 | 0.734 | 0.72 | 0.792 | 0.814 | 0.147 | 0.861 | 0.777 | 0.707 | 0.882 | 0.792 | 0.23 |

Abbreviations: AUC, area under the receiver operating characteristic curve.

Note: This table provides detailed performance metrics for the ten machine learning algorithms evaluated in this study, including the area under the receiver operating characteristic curve (AUC), accuracy, sensitivity, specificity, F1-score, and Brier score. Results are reported separately for the training cohort (n=291), internal validation cohort (n=125), and temporal validation cohort (n=233). These metrics provide the empirical basis for hyperparameter tuning and model selection, complementing the comparative overview presented in the main text.
